# A Scoping Review of Barriers and Facilitators Affecting the Lives of People With Disabilities During COVID-19

**DOI:** 10.3389/fresc.2021.784450

**Published:** 2022-01-25

**Authors:** Samantha Croft, Sarah Fraser

**Affiliations:** Interdisciplinary School of Health Sciences, Faculty of Health Sciences, University of Ottawa, Ottawa, ON, Canada

**Keywords:** disability, COVID-19, pandemic, inclusivity, barriers, facilitators, social participation, rehabilitation

## Abstract

**Purpose:**

This scoping review aimed to identify the barriers and facilitators to everyday activities and social participation of people with a disability (PWD) during the first wave of the COVID-19 pandemic.

**Methods:**

The search terms (disability and COVID-19) were used in four databases: CINAHL, Medline (Ovid), EMBASE and Web of Science. The search conducted from January 2019 to September 22, 2020, identified 465 peer reviewed articles and abstracts and were screened in Covidence software. Studies were included if they had the terms “COVID-19” and “disability,” were published in English, and specifically examined how COVID-19 impacted the daily lives of PWD. Exclusion criteria included: disability as a symptom or result of COVID-19, the health outcomes when PWD acquired COVID-19, disability leave for someone who is sick and the risk of acquiring the disease for PWD. 74 articles met the inclusion criteria and were analyzed via data charting. Charting began with existing barriers and facilitators identified by the World Health Organization and new barriers and facilitators, that emerged from the texts were added during this process.

**Results:**

The barriers that emerged included: access to information, ease of communication, financial impacts, mental health impacts, access to essential services, physical safety, educational challenges, and changes to care and rehabilitation. Significant facilitators included: changes to care and rehabilitation, new innovations, social and familial support and inclusive policy measures.

**Conclusion:**

COVID-19 exacerbated existing challenges in the lives of PWD and raised new quality of life concerns. Findings also demonstrate that policy makers, health care professionals and others continually support PWD in times of crisis.

## Introduction

In December 2019, doctors in Wuhan, China identified a cluster of pneumonia cases that were caused by the novel coronavirus SARS-Co-V2 (1). Despite efforts to contain the virus and its consequent disease COVID-19, the World Health Organization (WHO) declared that the outbreak had reached pandemic levels by March 2020 (2). Six months later, the WHO had reported nearly 33 million cases, alongside almost one million deaths worldwide, with both statistics still climbing rapidly. As with most health and humanitarian crises, certain vulnerable populations are more susceptible to adverse outcomes during this pandemic. The United Nations (UN) definition of disability inclusion is the meaningful participation of people with disabilities (PWD) in all their diversity, the promotion of their rights and the consideration of disability-related perspectives (3). The UN stated that PWD are particularly disadvantaged by the socio-economic and health consequences of the COVID-19 pandemic (4). This is not surprising, as PWD have regularly experienced a variety of access and inclusivity barriers (5). PWD across all socioeconomic circumstances struggle to receive enough financial compensation, equal job opportunity and inclusive care and rehabilitation (5). The UN has predicted that the COVID-19 pandemic has exacerbated aforementioned existing inequalities, in addition to proposing new challenges to PWD (4).

Nearly all countries have implemented stringent measures to mitigate the risk of COVID-19 spread, which commonly includes physical distancing policies, economic lockdowns and a rapid shift to virtual life, among other modifications (6). These changes have dramatic impacts on the lives of all individuals, but create unique challenges for PWD. For example, many PWD have health issues that require them to frequently attend in-person care appointments, many of which would have been cancelled or switched to virtual format at the beginning of the pandemic. These challenges will be explored at length in the results section. Even though the UN (4) and the WHO (7) have released documents outlining specific frameworks for governments to incorporate disability inclusion in their pandemic responses, a preliminary search through the literature conducted by Samantha Croft (SC, author) revealed that many inequities are not being addressed.

In contrast to the barriers and increasing exclusion of PWD in certain activity and participation situations (8), there is some evidence that individuals and corporations have found innovative ways address inclusivity barriers in the time of COVID-19. For example, a popular media piece at the beginning of the pandemic outlined how certain grocery stores were offering unique hours for “vulnerable” populations to shop, and soon after, many supermarkets worldwide implemented the same change (9). This suggests the pandemic may be highlighting the inequalities that PWD have been facing for decades, and may provide an opportunity for governments to address them and offer solutions (10). Overall, new findings are emerging that examine both the barriers and facilitators for the daily lives of PWD during this pandemic.

While it is important to address the health outcomes for COVID-19 cases, as well as the risk of acquiring the disease for PWD, the focus of this review is to examine how pandemic responses have affected the daily lives of PWD. While some studies have reported findings on how the daily lives of PWD have been affected by the COVID-19 pandemic (11, 12), to our knowledge, no review has been published that has compiled the aforementioned research to identify common experiences of activity restrictions and social participation limitations of PWD during the COVID-19 pandemic. This scoping review intends to fill this gap in the literature by answering the following research question: How have the pandemic responses affected the daily lives of PWD worldwide? The objective of this paper is to identify common inclusivity successes and failures of the COVID-19 pandemic response seen globally. Emphasizing these common themes has the potential to shape the future of inclusive policy-making, inform research on this topic, and maximize positive, inclusive approaches for PWD and their families and caregivers.

## Materials and Methods

Researchers Arksey and O'Malley define a scoping review as, a review technique that involves ‘mapping‘ the relevant literature in a field of interest (13). A scoping review is particularly suited for the exploration of broader and newer research topics (13) and therefore is appropriate to examine the COVID-19 pandemic and its impacts on PWD as this is a novel research area. Scoping reviews do not assess the quality of the studies included but are often used to: decide whether to proceed to a systematic review, summarize the and disseminate the research findings in a specific area and identify any potential gaps in the literature (13). The authors were guided by the methodological framework set out by Arksey and O'Malley (13), and the Preferred Reporting Items for Systematic reviews and Meta-Analyses extension for Scoping Reviews (PRISMA-ScR) (14).

**Figure 1:**
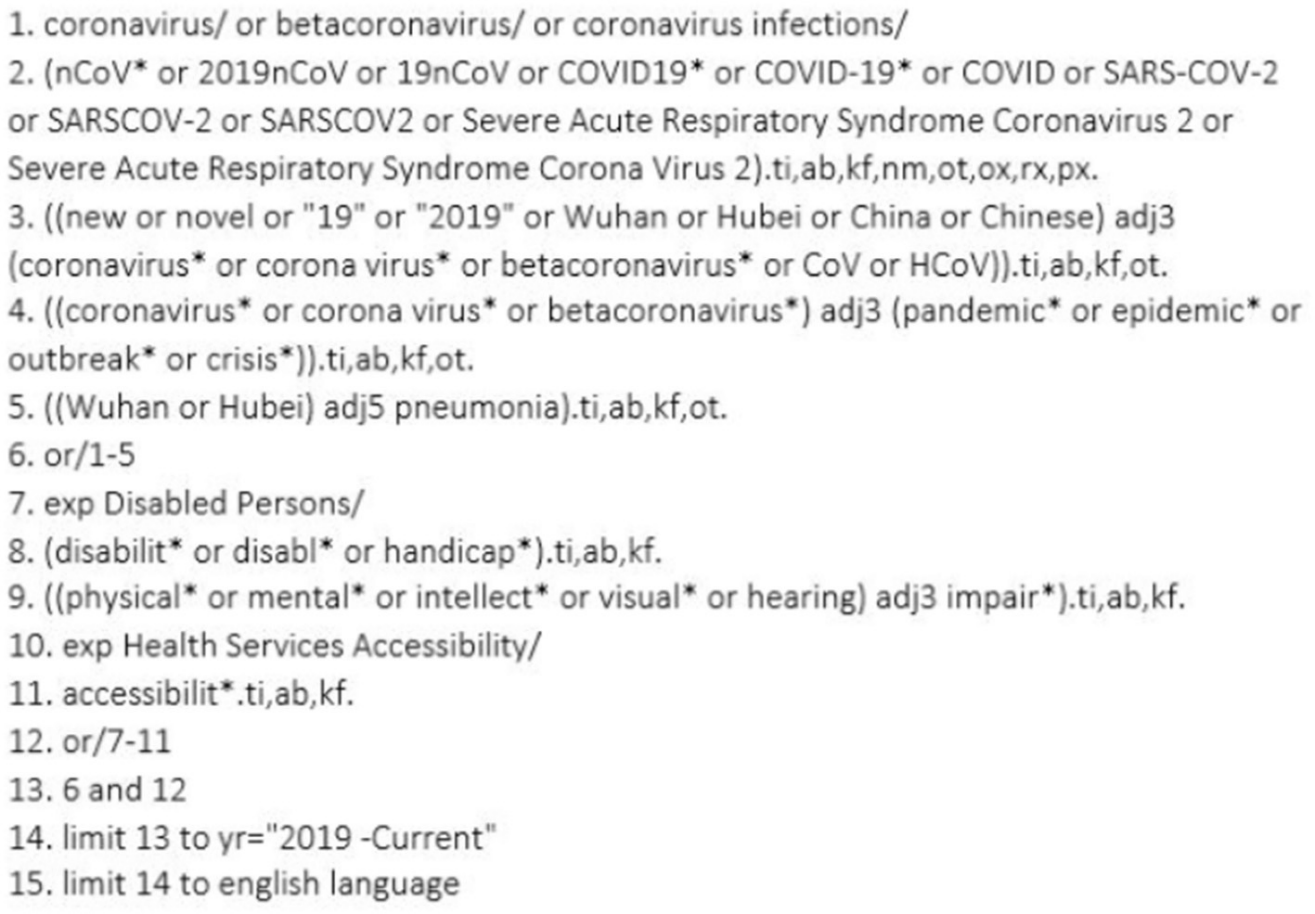
Full search strategy used on database Medline (Ovid). #1-5 are referring to articles containing coronavirus and all varying modifications to the term. #7-11 denote articles containing disability, accessibility and all varying modifications to the terms. Finally, #12 and #13 are a combination of the above concepts, while #14 and 15 are limiting the search by year and English language.

SC developed the search strategy alongside an experienced librarian in the Health Sciences department at the University of Ottawa, and the final search terms were confirmed by SC and Sarah Fraser (SF, author). The key search terms were “COVID-19” and “disability,” but they also encompassed the range of alternate vocabulary used to identify these topics to ensure all relevant research was included (see Figure 1 for full details).

### Inclusion Criteria

It is important to note that the authors of this paper, SC and SF, included intellectual/developmental, sensory, physical and psychological disabilities, as types of disability that are impacted by the current pandemic. Additionally, articles discussing PWD of any age, children, or adult, were included for analysis. Furthermore, for feasibility reasons, this study focused on contexts that would encompass the daily lives of PWD, such as community, home, rehabilitation, and educational settings. Articles published from January 2019- September 22 2020 were targeted, in order to ensure that only articles examining the novel SARS-Co-V2 virus were included. Furthermore, articles written in English were eligible for review as it is the only language fluently shared by both reviewers. Except for existing reviews on this topic, all types of published journal articles were eligible for review (i.e., opinion articles, commentaries, etc.).

### Exclusion Criteria

Articles were excluded if they discussed disability as a symptom or result of COVID-19, the health outcomes when PWD acquired COVID-19, disability leave for someone who is sick or the risk of acquiring the disease for PWD. We also omitted other review articles in the screening process.

**Figure 2:**
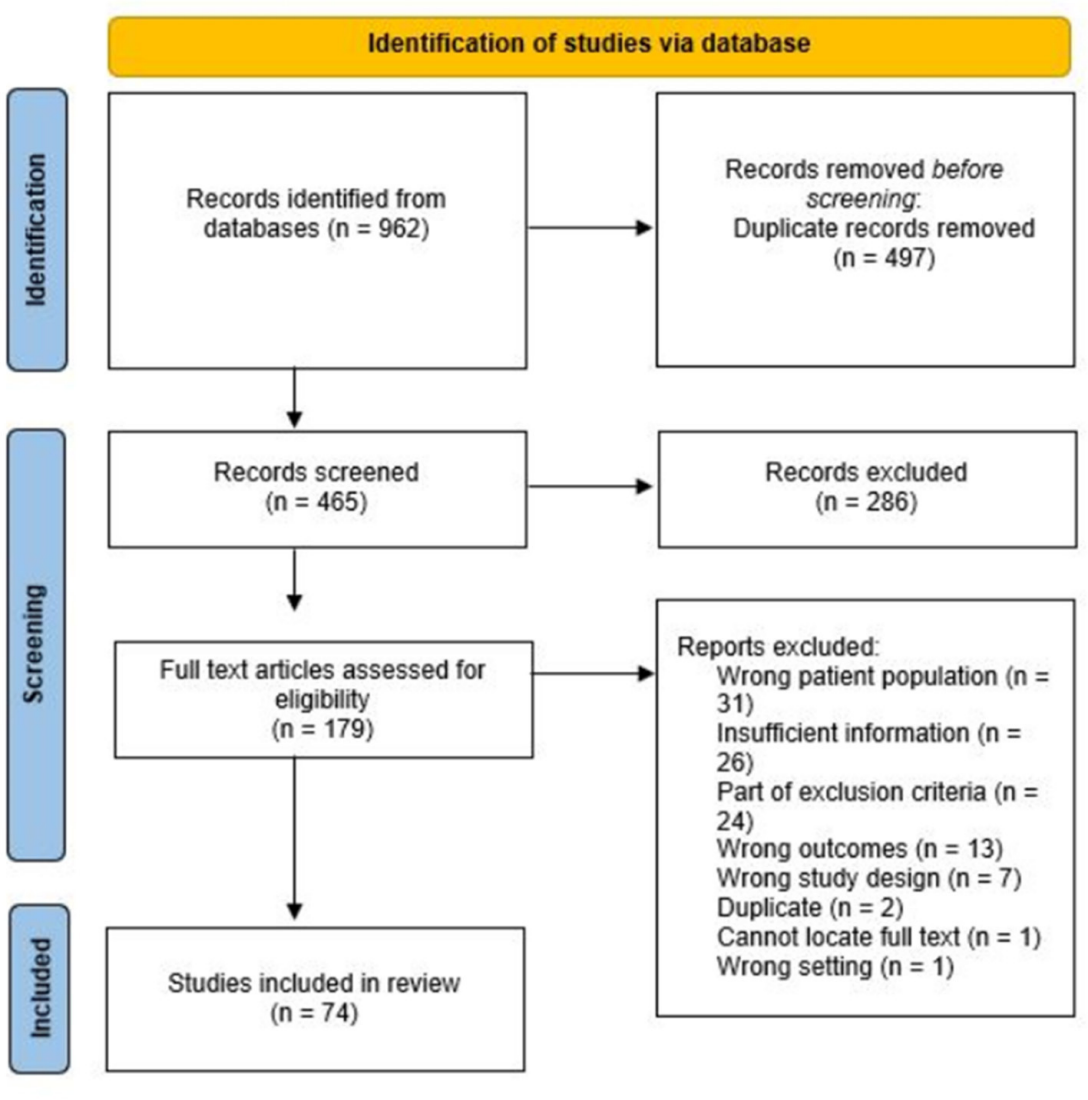
Scoping review flowchart.

The search was conducted on September 22, 2020 and the final search terms were used in the following databases: CINAHL, Medline (Ovid), EMBASE and Web of Science. After duplicates were removed in Covidence review software (15), 465 articles remained, which were then screened by title and abstract. Articles were retained if they supported the research question and inclusion criteria. Both SC and SF screened the title and abstracts of the first 200 articles, and any necessary discussion and conflict resolution was conducted at regular bi-weekly intervals. As there was 81% agreement between the raters and a Kappa of 0.6 after half the abstracts were screened, the remaining abstracts were screened solely by SC. Of the 465 articles, 179 were retained for full text screening. Articles were dismissed if their titles and abstracts did not conform to this research question (i.e., discussing the impacts of pandemic responses on the daily lives of PWD). The full text screening was conducted by both authors, which also included regular discussion and conflict resolution via video call. After careful consideration of the exclusion and inclusion criteria, 74 articles remained for thematic analysis (see Figure 2 for full details of the screening process). Specifically, 31 articles were excluded for discussing the wrong patient population (i.e., non-disabled individuals) and 26 articles were excluded for insufficient information regarding this study's research question. Further, the authors omitted 24 articles based on the exclusion criteria, (i.e., the risk of PWD acquiring COVID-19). Thirteen studies had wrong outcomes, for example measuring doctor errors when treating PWD. Seven articles were excluded for having the wrong study design (i.e., existing rapid scoping reviews). Finally, one study was set in hospitals, rather than in everyday settings for PWD, and the remaining three articles were excluded because they were either duplicates that were initially missed, or the full text could not be located.

**Figure 3:**
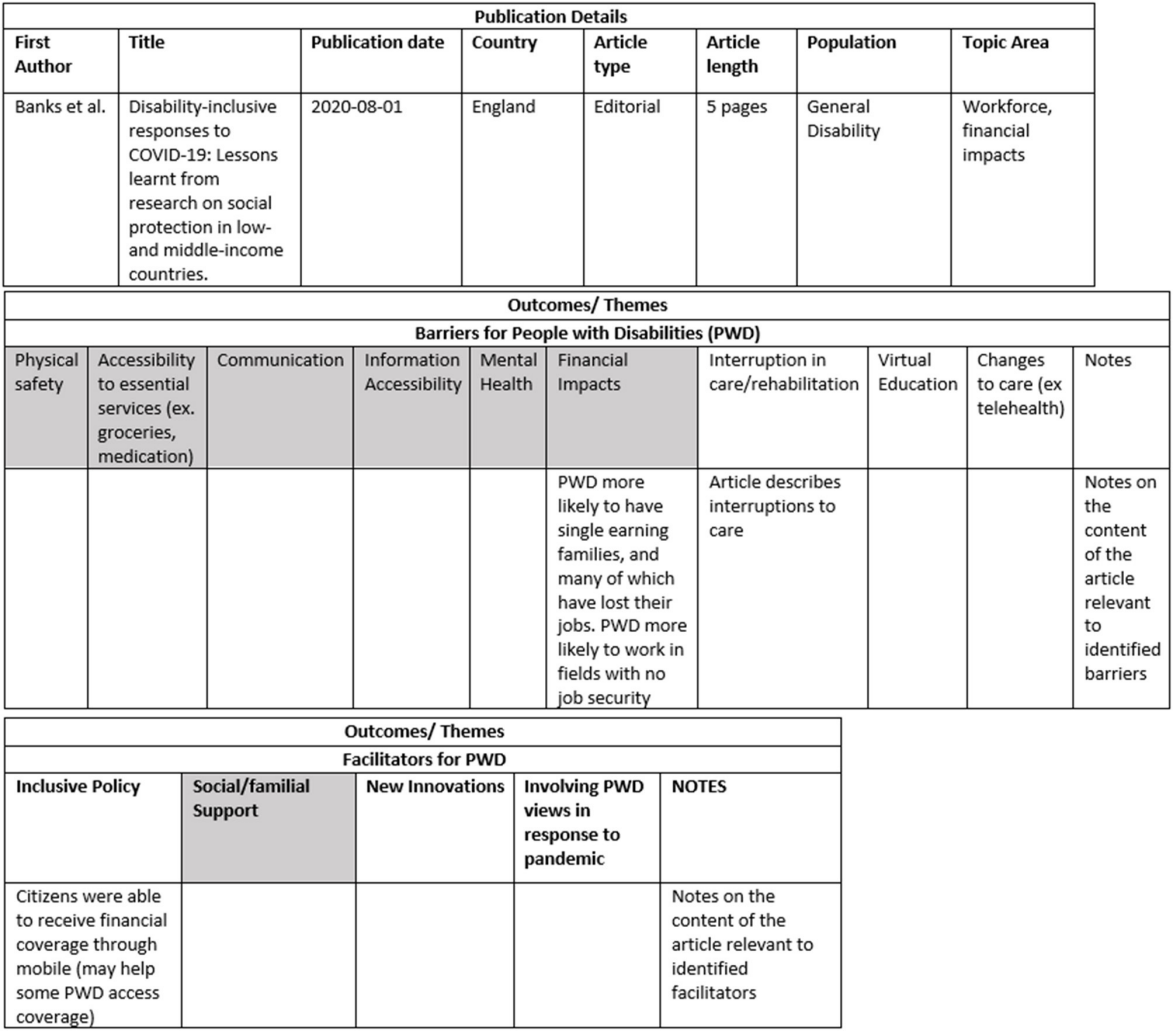
Data-charting template used by the authors. Data charting included three sections: Publication details, Barriers for people with a disability (PWD) and Facilitators for PWD. Barriers and Facilitators in grey in the table represent existing barriers identified by the WHO and additional barriers/facilitators that emerged from the data in are white. This figure includes the data charting for one article by Aishworiya et al. (16), and notes are not included in this example of the data charting as they are lengthy.

The data-charting template was created by author SC and refined by SF. Barriers and facilitators were created a priori based on results from preliminary searches through the literature, specifically from the WHO report “Disability considerations during the COVID-19 outbreak” that was released early in the pandemic (7). However, the barriers and facilitators were also updated iteratively as new themes emerged from the included articles. The authors chose to extract general data such as main author name, article type and length, country of origin, population of study, and key topics covered. Additionally, SC extracted thematic and sub-thematic data, as well as significant notes and quotations from the articles. An example of the data chart used can be found in Figure 3. Please note only the data from one included article is shown as an example. Finally, SC conducted the summarization, collation and report of the results, using the information she extracted and charted, and this was deliberated and discussed with SF.

## Results

### Article Characteristics

**Figure 4:**
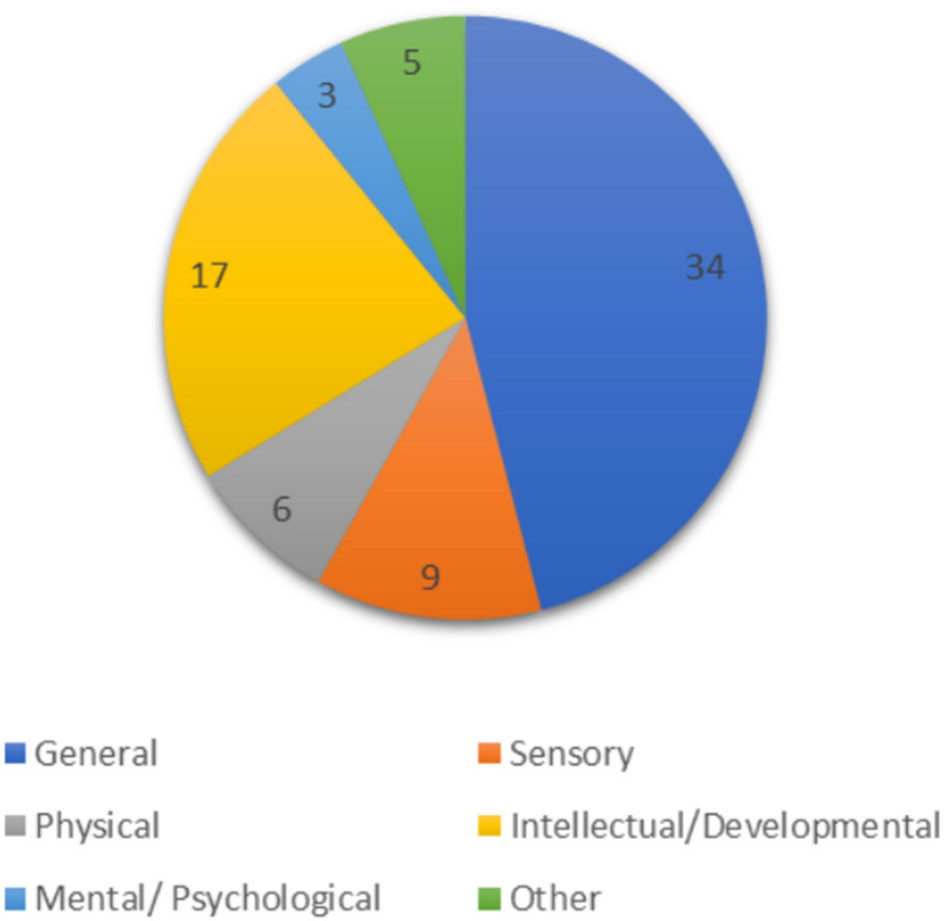
Publications per disability type.

After the full text screening process, the authors were left with 74 articles for extraction and analysis. Of these, 55 articles (74.3%) were literature such as editorials, opinion and commentary articles. The remaining 19 articles (25.6%) were observational studies, including cross-sectional, case and framework analysis studies. The articles examined barriers and facilitators for PWD from a wide variety of countries, the most common being the USA, the United Kingdom and Brazil. However, many included commentaries and opinion articles did not specify a country of study, which is why there is no detailed data on country of origin for the included articles. When examining the type of disability, 34 articles (45.9%) did not specify a disability type, but rather discussed PWD in general. Furthermore, nine articles (12%) focused on individuals with sensory impairments, including populations who are deaf or hard of hearing (DHH), visually-impaired or blind. Additionally, 17 articles (22.9%) discussed how COVID-19 has impacted individuals with intellectual and developmental disabilities (IDD), such as autism spectrum disorder (ASD). Meanwhile, three articles (4%) detailed mental and psychological disabilities, such as obsessive-compulsive disorder (OCD). There were six articles (8%) included that discussed physical disabilities, including spinal cord injuries (SCIs), chronic pain conditions and osteoarthritis. Finally, there were five articles (6%) pertaining to a variety of classifications (i.e., diseases of the nervous system, congenital malformations of the nervous system, etc.), such as Parkinson's disease (PD), multiple sclerosis (MS), and spina bifida (17). This category will be referred to as “Other” (see Figure 4 for a visual representation of this data).

### Important Themes That Emerged From the Data

**Figure 5:**
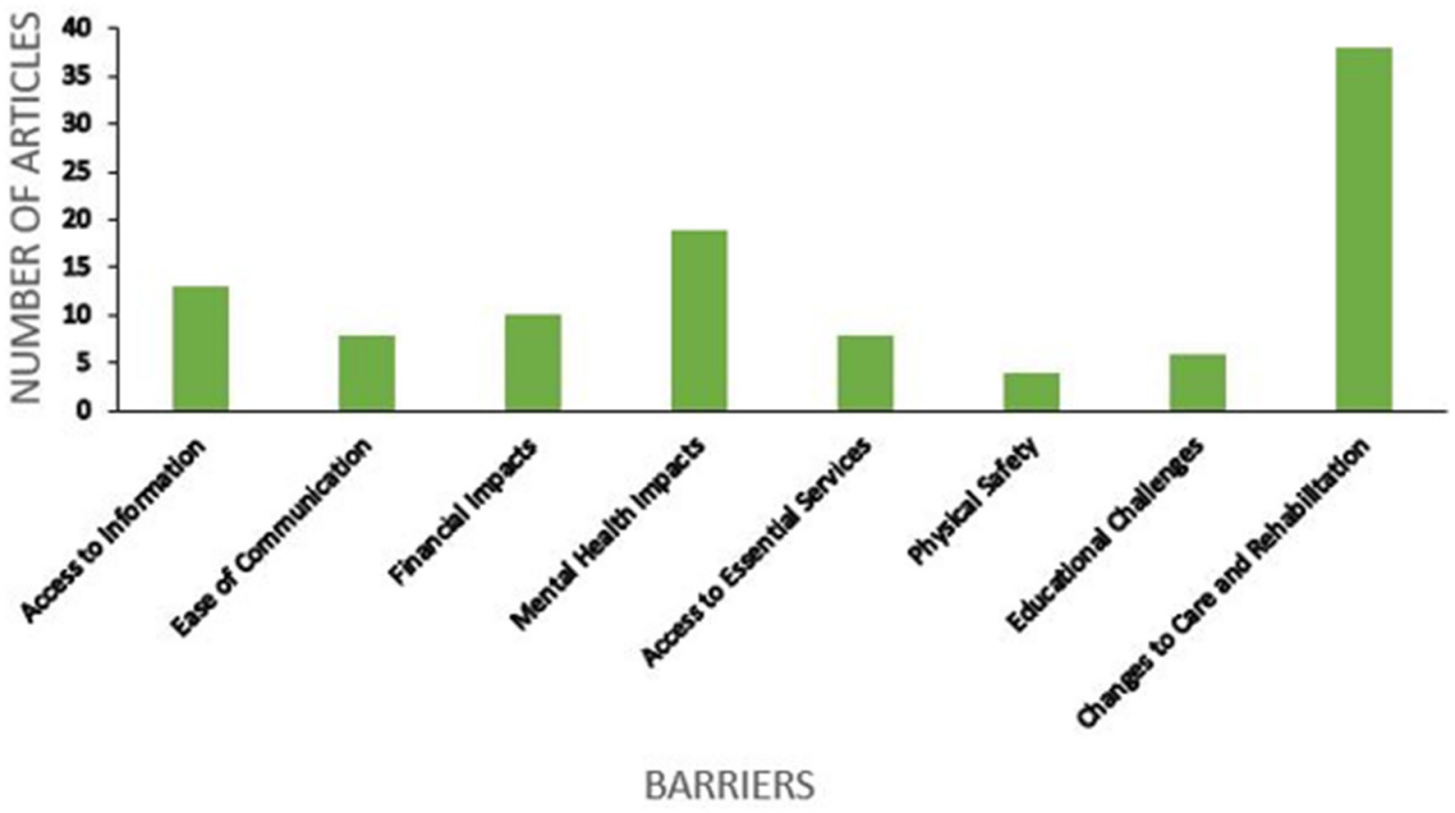
Number of articles per barrier.

**Figure 6:**
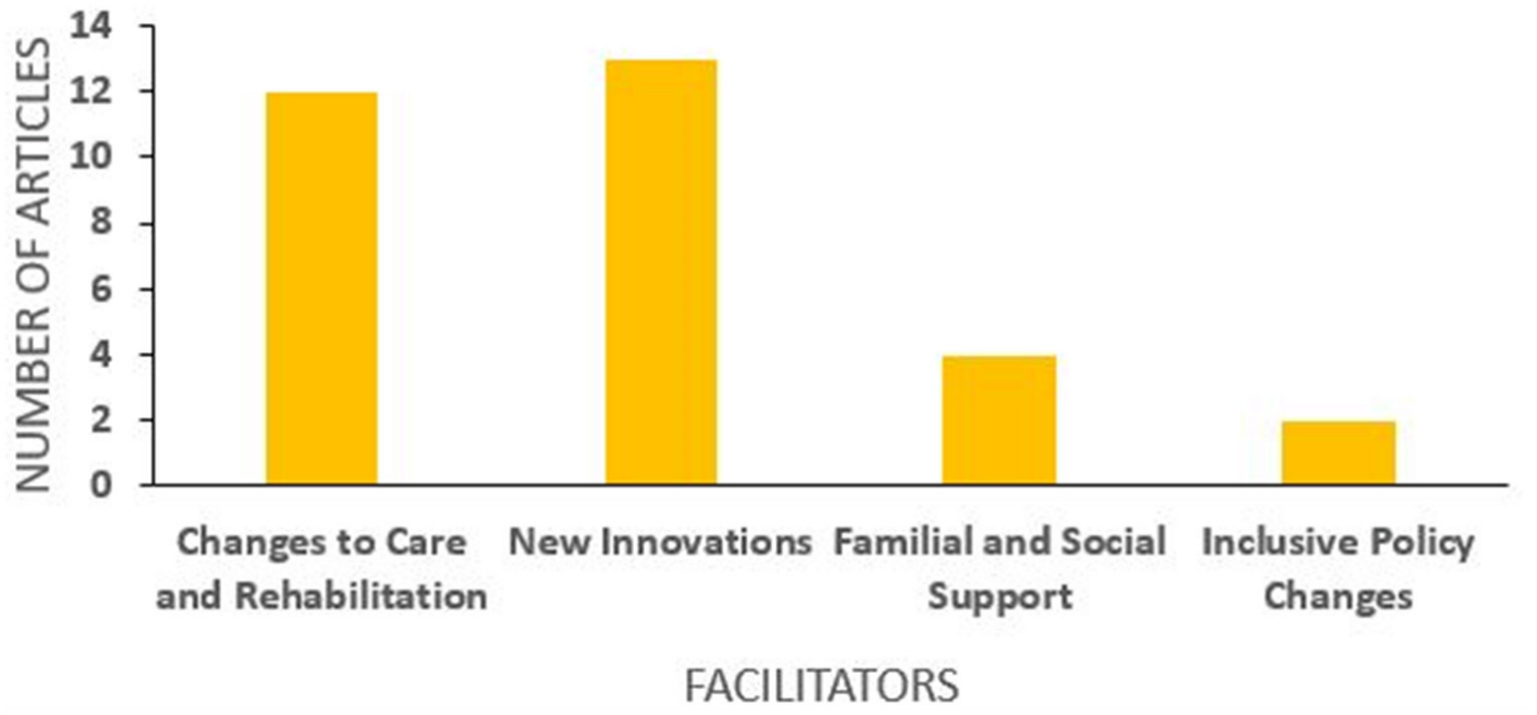
Number of articles per facilitator.

The findings were first categorized into two main themes: barriers and facilitators for the lives of PWD during the COVID-19 pandemic. These were then subcategorized into several subthemes that were commonly presented in the articles. The barriers noted included: (a) access to information, (b) ease of communication, (c) financial impacts, (d) mental health impacts, (e) access to essential services, (f) physical safety, (g) educational challenges, and (h) changes to care and rehabilitation. Significant facilitators included: (a) changes to care and rehabilitation, (b) new innovations, (c) social and familial support and (d) inclusive policy measures. It is noteworthy that one subtheme, changes to care and rehabilitation, is described as both a barrier and facilitator. This will be explored at length in the discussion (see Figures 5, 6 to visualize how many articles discussed each theme. Please note that many articles discussed multiple themes).

#### Barriers for PWD During the Pandemic

*(a) Access to Information:* In a crisis such as the COVID-19 pandemic, messages from the government and international organizations such as the WHO must reach the public quickly to keep the citizens informed (18). This often leads to a lack of attention paid to those who may have alternate communication needs, despite the UN's Convention on the Rights of Persons with Disabilities specifying that access to information is vital during humanitarian crises (19). Therefore, it is not entirely surprising that the findings revealed that many PWD had difficulties accessing public health messaging regarding COVID-19 due to a lack of proper accommodation, such as subtitles and sign language interpreters (19–25).

The results indicate that information regarding COVID-19 has often been inaccessible to individuals with IDDs specifically (26, 27). For example, Fernandez-Diaz et al. (28) found that the WHO website rated poorly on an operability scale, meaning that it was difficult to navigate and find relevant information within the website for people with IDD.

Individuals with visual disabilities or blindness have also faced challenges retrieving accessible COVID-19 information (27–29). A study by Fernandez-Diaz et al. (28) revealed that while the WHO's website did have some alternative text available, many pages had errors, or did not have any alternative text available. Furthermore, the website rated poorly on color contrast, which can aide visually-impaired individuals with website navigation (28). Sabatello et al. (29) noted that COVID-19 information is frequently portrayed in infographics that are very difficult for visually-impaired individuals to read. Finally, Guidry-Grimes et al. (27) identified the need for audio descriptions to increase inclusion during press conferences.

This situation is similar for people who are DHH, who experience difficulties understanding information from government press conferences unless subtitles or sign language interpreters are utilized (29). Unfortunately, Yap et al. (19) discovered that only 65% of publicly-available pandemic-related briefings that occurred in low- and middle-income countries, and 0% of that of international organizations such as the WHO offered a sign language interpreter during the first months of the pandemic. Additional articles note similar findings in other nations, such as China (27, 30, 31).

*(b) Ease of Communication:* New COVID-19 policies, such as virtual health care and mask mandates, have implemented barriers for certain PWD to communicate. Several included articles note that it is extremely challenging for many DHH individuals to communicate in public while wearing masks, as it directly impedes one's ability to lipread or hear what is being said (32–35). Medical student Isabelle Williams (36), who is DHH, states that her ability to communicate on the job is severely limited by masks, and she is troubled by the lack of guidance regarding how to wear both full personal protective equipment (PPE) and an auditory aide at work. Furthermore, for PWD who are attending school or care appointments virtually due to the current social distancing protocols, effective communication has been reported as nonexistent where no accommodation is provided (37–39).

*(c) Financial Impacts:* In general, PWD are more likely to have financial disadvantages, due to underlying factors including the increased cost of living for PWD, the inaccessibility of certain jobs, and expensive health concerns (6, 40). The financial disparity between disabled and able-bodied individuals is exacerbated by the COVID-19 pandemic (26). PWD are often a part of single-income families, have jobs with lower wage and/or security, and are often the first to lose their jobs during budget cuts such as those seen during COVID-19 (41–43) and face limited financial supports during the pandemic (44).

While some countries have offered financial support to PWD, these packages were often insufficient (45–47) and/or not provided to those who already received a disability pension (47).

There are also other inequities regarding financial wellbeing during the pandemic. A commentary published in the USA states that among people with acquired brain injuries (ABIs), women and members of the lesbian, gay, bisexual, transsexual, queer plus (LGBTQ+) community are more likely to have negative financial impacts due to the pandemic (48).

*(d) Mental Health Impacts:* The COVID-19 restrictions have created negative mental health impacts on nearly all societal groups; however, the literature suggests that PWD may be facing mental health issues at a greater rate (16, 35, 44, 49–52). Boldrini et al. (44) note that Italian citizens with disabilities have experienced an amplification of isolation, loneliness and a perceived lack of support since the pandemic began. Children with IDD, such as ASD, are particularly vulnerable to adverse mental health outcomes of the lockdown (16). As many children with IDD rely on structured programs, routines and clear expectations set out for them for mental health benefits, the disruption of everyday life is difficult for them to accept and comprehend (16, 35, 49, 51, 52). This disruption of routine and consequential confusion has led to an increase in anxiety, distress and even a deterioration in development and intellectual progress for children with IDD (21, 52–54).

Where programs have continued, the increase of preventative measures, such as staff wearing full personal protective equipment (PPE) and the ban of visitors to care centers, have also created decreased mental health outcomes for individuals with IDD (50). It is also more difficult for people with IDD to utilize social media for social connection, which exacerbates their feelings of isolation (45). Furthermore, the United Kingdom initially implemented a COVID-19 policy that only allows individuals to go outside once per day (55). Children with IDD faced mental health impacts from this sudden containment and restriction, and the government has since allowed increased time outside if it benefits individuals' mental health (55). Overall, in the United Kingdom, mental health decline has resulted in increased requests for psychotropic medication by caregivers of people with IDD in attempt to handle their behavioral challenges that have been exacerbated by the COVID-19 lockdowns (21).

The physical distancing and program closures related to COVID-19 have also affected people with physical disabilities. A French study conducted by Cacioppo et al. (11) on the mental health of children with physical disabilities revealed that more than half of the participants had increases in behavioral issues and sleeping difficulties since the lockdowns were implemented. Furthermore, individuals with chronic conditions are more at risk of acquiring mental health conditions such as anxiety and depression, even during normal circumstances (56). Finally, a survey implemented by Azzam et al. (57) also revealed that individuals with irritable bowel disease experienced significantly decreased body image scores over the course of the pandemic.

Additionally, the COVID-19 lockdowns have intensified the symptoms of people who live with mental health disabilities, and in some cases, increased the risk of suicide and severe psychiatric morbidities (23). For individuals with obsessive compulsive disorder (OCD), the government recommendations to adapt to increased safety and hygienic precautions may trigger an exacerbation of one's obsessions and compulsions, specifically hand-washing and cleaning, and may contrast what their doctor is instructing them to do to treat their OCD (58). Similarly, many Japanese citizens with anorexia have experienced a worsening of their condition due to anxieties surrounding the pandemic (42).

A cross-sectional study by Umucu and Lee (59) examined coping strategies of PWD during the pandemic and the consequent impacts on mental health status. One of the most common coping strategies used by the participants during the pandemic was self-distraction, which is correlated with increased stress levels, along with denial, substance use, behavioral disengagement, venting, planning, religion, and self-blame.

*(e) Access to Essential Services:* During the COVID-19 crisis, many PWD worldwide have experienced complications when accessing essential needs such as food and medication (14, 23, 31, 60, 61). A British study conducted by the Chronic Illness Inclusion Project revealed that 86% of respondents with chronic illnesses reported that the pandemic has had a negative effect on their ability to access food and essential goods (13). Commentaries by researchers Jumreornvong et al. (61) and Kuper et al. (23) have noted similar observations in the USA and United Kingdom, respectively. A worldwide study by Cheong et al. (60) found that nearly half of all participants with PD experienced barriers to accessing their regular medications, usually due to transportation interruptions and financial issues. These occurrences were much more frequent in low- and middle-income nations (60). Similarly, Qi and Hu (31) reported that individuals with MS in China were undergoing difficulties when purchasing medication and essential supplies due to public transportation closures.

When examining access to food and medication, some PWD experienced challenges when utilizing virtual grocery ordering services (33). While certain vulnerable populations were offered financial support for essential supply delivery in the United Kingdom, some PWD were not eligible (33). In other locations within the United Kingdom, essential supply delivery was canceled altogether to reduce the risk of disease spread, which posed challenges for PWD who usually rely on the service (20).

*(f) Physical Safety:* The COVID-19 pandemic has threatened the physical safety of PWD in several ways (33, 40, 48, 61). Firstly, the stress surrounding stringent public health measures and financial pressures due to COVID-19 may trigger the commencement or increased intensity of domestic abuse toward a disabled family member (40). Furthermore, there may be an increase in domestic violence simply because a person with a disability is spending more time at home with their abuser (61). Lund (40) also notes that it is much more difficult for victims to report this violence since PWD no longer see trusted contacts they confide in, in-person organizations were closed or offering virtual care in which a perpetrator may be in earshot within the home. This produced an environment where it is nearly impossible for PWD to report abuse (40). Furthermore, among individuals with ABIs, women and members of the LGBTQ+ community are more likely to experience interpersonal and domestic violence (48).

Additionally, certain physical safety barriers arise when PWD cannot ask strangers for assistance due to physical distancing mandates (33) or are waiting for necessary equipment or assistive devices to be repaired (40). For example, debris and other hazards on the sidewalk become much more dangerous for visually-impaired individuals when they are unable to ask for a stranger's aid for fear of contracting COVID-19 (33).

*(g) Educational Challenges:* Many educational settings worldwide transitioned to a wholly or partially virtual method for a period of time during the COVID-19 pandemic, which generated issues for PWD (37, 39, 47, 52, 62, 63). Few schools accommodated students who are DHH, even though they face challenges with virtual communication in the absence of accommodations such as a sign language interpreter (37). Furthermore, virtual education platforms are often inaccessible for people with physical, sensory or intellectual disabilities who normally have structured and individualized educational plans involving educational assistants, interpreters and other services, have not had these accommodations applied during virtual education use (47, 52, 62). Online schooling removed many educational aspects that PWD benefit from. For example, without the recreational activities and exercise that children with ASD usually access at school, they can get irritated, confused, and can even demonstrate stunted development (52). Furthermore, one Brazilian university professor notes difficulties teaching students with IDD challenging anatomical details without the practical use of cadavers, 3D models, and other helpful applications (63).

*(f) Changes to Care and Rehabilitation:* Many of the included articles across several countries such as the United Kingdom, Australia, Italy, France and Turkey note that PWD have experienced interruptions in their regular rehabilitation (14, 16, 20, 22, 41, 45, 50, 64–67). This occurrence has been noted across many disability types, including psychological and intellectual and developmental (64, 67).

In particular, 65% of PWD reported disruption to care during the pandemic, and more than half reported worsening health outcomes (13). Among children with IDDs, 30% within the USA and 50% outside the USA lost *all* rehabilitative services (67). A French survey revealed that 77% of children with physical disabilities experienced cancelled or postponed medical consultations during the pandemic (12). A study by Negrini et al. (68) estimated that in Europe, more than one million people per day were being denied their rehabilitation services in acute, post-acute and outpatient settings.

Closures of pain management clinics have had adverse impacts on individuals who are disabled by chronic pain and rely on the clinics for appropriate treatment (69). This has led to the increase of harmful or inappropriate pain interventions such as the abuse of nonsteroidal anti-inflammatory drugs, opioids and illegal substances (69). Secondly, for children who are having the first signs and symptoms of a disability during the pandemic, proper assessment, diagnosis and initial care are being delayed due to perceived non-urgence of the situation (22, 70). Tied closely to the mental health subtheme, individuals with IDDs can suffer from extreme mental distress as a result of disruption in regular care routines (51). These individuals, as well as those who are DHH or visually-impaired, may experience an intensification of their disabilities when being denied from the care they rely on for progress (39, 51, 71). Moreover, despite the importance of dental care, there is evidence that vulnerable populations have been neglected during the pandemic (72, 73). Dental workers acknowledge the increased anxieties surrounding the pandemic and recognize that going to the dentist is another cause of anxiety (72).

Individuals with specialized rehabilitation needs also suffered from the closures, as it was hard to access proper professional expertise (35, 44, 74). For example, Italian citizens with SCIs or a rare condition called Charcot Marie Tooth Disease experienced difficulties acquiring a care plan, as the few experts in the field were not accepting patients (44). In Austria, many individuals needing osteoarthritic knee and hip replacements were forced to postpone their surgeries, which ultimately decreased joint agility, physical function and activity levels in the individuals (74). Finally, the lack of blood donation drives due to pandemic closures has had major implications for populations who rely on regular blood transfusions, such as individuals with thalassemia major (35).

Additionally, rehabilitation services may be difficult to access even if the services are open. For example, care may be abruptly halted if support workers get sick or must tend to a sick family member (75). When these situations have occurred in China, agencies and governments have had difficulties finding last-minute replacements, which left PWD without essential care (31). Unfortunately, some countries have witnessed cases where no back-up care was provided, and PWD were left alone without care for several days, with one person in China even dying due to neglect (30, 31, 76). Furthermore, in a country such as Iran, where nearly all rehabilitation services are offered in urban settings and public transportation was temporarily shut down due to COVID-19, PWD living in rural settings had no way of accessing necessary services for their care (46).

A large portion of health care and rehabilitation has transitioned to telehealth, using video and audio calls to conduct treatment. The included articles reported that a significant disadvantage of telehealth is the need for high-speed internet (38). Families without technological applications such as computers and a strong internet signal risk being excluded by new telehealth services (16). Telehealth also poses significant barriers for individuals with sensory disabilities if a sign language interpreter is not available (32, 34). This type of platform may not be useful or appeal to certain PWD (i.e., cognitive impairment, impulse control, psychosis) (60). It is also challenging for health care professionals to utilize telehealth to prescribe painkillers to individuals living with chronic pain, as this often requires in-person testing (54, 69). Furthermore, audio tests conducted to monitor and diagnose individuals with DHH require perfect audio quality, which is rare via telehealth, making the service inappropriate for use of hearing impairment diagnoses and testing (70). Kolakowsky- Hayner and Goldin (48) note that women and members of the LGBTQ+ community with ABIs are more likely to lack the technological access required to attend a telehealth appointment.

It is true that family members of PWD can ease many barriers of telehealth (77) such as setting up the person in front of the computer, and conducting small assessments for the physician that require touch. However, this also has its drawbacks. Firstly, many individuals do not have a reliable and engaged familial support system, and it is unfair for this to be a requirement to access rehabilitation (78). Kolakowsky- Hayner and Goldin (48) also state that women and members of the LGBTQ+ community with ABIs are less likely to have the familial support often required to attend a telehealth appointment. Longo et al. (78) state that even if parents are willing to take over therapy and rehabilitation at home, this can put an additional stress on the family. Family members may be asked to help conduct tests or make specific observations, which may be inappropriate and risky, as the caregiver may not be trained in medicine or not understand the scientific implications (45). For PWD who need technological help from others in order to use telehealth, the possibility of privacy and confidentiality during care is eliminated (29, 40).

#### Facilitators for PWD During the Pandemic

*(a) Changes to Care and Rehabilitation:* Despite the several aforementioned disadvantages of telehealth, it is important to note the benefits it provides, as well (77). The pandemic situation makes safe face-to-face care relatively impossible, and thus, it is important to note that a key benefit of telehealth is that it provides an opportunity for safe continuity of care (61). Another commonly-cited advantage of telehealth usage for PWD is the increased ease of access (38, 64). Whereas, traveling to and from a clinic can often be time-consuming and inconvenient, specifically for PWD, telehealth can be accessed from the comfort and convenience of home (46, 60). Telehealth also gives the opportunity for health care professionals to observe the client in their own home, which may lead to greater understanding of an issue and easier ability to propose a solution (45).

Telehealth is reported to be an appropriate method of care for several disability populations. For example, Dorsey et al. (79) state that PD possesses very characteristic symptoms that can usually be diagnosed visually, which makes telehealth perfectly suitable in this case. Furthermore, there are a variety of smartphone apps (i.e., Doctot & CloudUPDRS) that can make telehealth more efficient for individuals with PD (80). Miele et al. (80) also outlined several other useful virtual strategies for patients with PD, such as the NMS Scale to examine non-motor symptoms, and Lift Pulse (Lynx Design, National Institutes of Health, Bethesda, Maryland) to monitor resting tremor.

There are similar virtual tools available for patients with MS. Researchers Moccia et al. (81) specifically recommend using the Patient Determined Disease Steps program and the MS Impact Scale before a telehealth consultation to assess the patient's state. Furthermore, doctors and patients have had success using neurological tests and the oral version of the Symbol Digit Modalities Test during telehealth to analyze disease progression (81). Health care professionals have also noted effective use of telehealth for evaluation of muscle strength, balance, fatigue and stamina for individuals with neurodevelopmental disabilities (82). Telehealth has also been shown to be beneficial in treating and assessing pediatric feeding disorders, behavioral issues and language delay during ASD (83).

*(b) New Innovations:* The COVID-19 pandemic posed unprecedented issues to PWD that required novel solutions. As Bruder (84) states, the pandemic gave individuals “a chance to demonstrate the resilience of the human spirit when faced with adversity and uncertainty.” Around the globe, a variety of virtual leisure programs have been offered throughout the pandemic in order to help PWD exercise, socialize and keep busy while at home. Exercise programs were developed that aimed to mitigate the adverse outcomes of increased isolation and sedentary routines (85). These virtual exercise programs were offered by personal trainers, yoga instructors, and a variety of other specialists and may even offer a more accessible, inclusive and convenient way for PWD to exercise compared to traditional in-person gyms and classes (86). A notable case study by Lai et al. (87) applied a virtual reality exercise technology to children with spina bifida for use in their homes. The researchers reported that the participants experienced increased motivation, calorie expenditure and sense of accomplishment while using the technology (87).

There were also technologies that emerged during the pandemic that facilitated communication for individuals with sensory impairments. A simple yet efficient solution was the creation of clear masks so that individuals who are DHH could lipread (32, 34). Other innovations for DHH individuals include communication boards, automatic speech recognition platforms and virtual interpreters (34). Furthermore, Martinez et al. (88) developed a device for blind individuals that will give them a sound warning when they are within six feet from another person, in hopes of guiding them through physical distancing restrictions. Blindfolded and blind individuals using the prototype both reported successful results (88).

Included articles also reported a variety of other creative methods to increase inclusivity toward PWD, while remaining physically distanced and safe. Firstly, the National Autistic Society offered virtual movie and craft nights to mitigate the adverse effects of disrupted routines on individuals with ASD (55). Other inclusive innovations include special grocery store hours for vulnerable populations, and Zoom calls hosted by Delaware's Developmental Disabilities Services to provide relevant information pertaining to the COVID-19 pandemic and disability to families (26, 76). Innovations were also implemented in educational institutions. For example, certain schools offered specialist teachers and one-on-one virtual calls with the professor to solve any pertinent issues (39, 62). Finally, the Dutch program DigiContact was in place pre-pandemic to allow individuals with IDD who live independently to access virtual support 24/7 (89). As the use of this program was significantly increased at several points throughout the pandemic, it is clear that DigiContact facilitated access to any necessary online support for these individuals (89).

*(c) Familial and Social Support:* Since care, education, and work are occurring mostly from home, many PWD are able to spend more time with their families, which has increased quality of life for PWD in many circumstances (51, 53). One study by Neece et al. (51) revealed that many families with disability appreciated a slower pace of life, and enjoyed the increased opportunity for sleeping, relaxing and meditating. Finally, the use of telehealth sometimes offers the opportunity for parents to be more involved in the care of their child with a disability. In this way, some health care professionals are noticing an increase in enthusiasm and engagement in rehabilitation through this method compared to normal face-to-face care, during which the parents are usually less involved (90). PWD have also pursued extra social support, increasing their use of social media to connect with others and overcome negative aspects of isolation (64).

*(d) Inclusive Policy Measures:* A study by Sakellariou et al. (47) examined how disability-inclusive new COVID-19 policies have been in four South American countries: Peru, Brazil, Argentina, and Chile. Peru was the only country studied that passed COVID-19-specific legislation protecting PWD's rights to equal education, employment and health care during the pandemic, explicitly citing the United Nations Convention on the Rights of Persons with Disabilities (47). The Peruvian government implemented measures to guarantee the wellbeing of PWD, including monitoring their access to care and ensuring their protection from any potential violence. Peru, as well as Argentina, and Brazil began to offer remote registrations for financial support for PWD. These three nations, as well as Chile also increased the financial support that certain PWD could receive during the first months of the pandemic (47). Additionally, Argentinian citizens with a disability were automatically re-registered for financial support during the COVID-19 pandemic. Furthermore, the Chilean government ensured that PWD were able to receive care by granting them special privileges, including a permit to visit care providers, and the ability to be accompanied by a caregiver during appointments. Similarly, Argentina, Chile and Peru all introduced policies that allowed PWD and their caregivers to go for walks without special permission and/or ensured caregivers could attend work, even if the area was under strict quarantine measures (47). Finally, a study by Banks et al. (41) reported that Georgia, Mexico, Mongolia, Lesotho, Tunisia and São Tomé and Principe plan to implement new or expand existing financial assistance programs for PWD in their COVID-19 response. Further, Gambia, Morocco and Togo now offer cash transfer distribution through mobile applications, rather than in-person, which would increase accessibility for some PWD (41).

## Discussion

This scoping review is unique because it focused on how the daily lives of PWD have been affected by COVID-19 protection measures, rather than examining COVID-19 treatment and risk of PWD acquiring the disease (91, 92). Several important barriers and facilitators present in COVID-19-infected countries were revealed, which highlight certain areas of concern for the social participation of PWD and potential avenues for future research.

Many of the themes that emerged in the findings were common issues that PWD faced prior to the pandemic that were exacerbated by pandemic conditions (5). For example, PWD still experienced inaccessibility to adequate health care services and negative financial impacts during the first wave of the pandemic, and there was evidence that the occurrence of these situations was increased due to COVID-19 (12, 16, 20, 22, 26, 41–47, 50, 64–67). In addition, new issues arose for PWD during the pandemic, such as: access to accurate COVID-19 health information, virtual education, and health care challenges. However, certain topics that were anticipated to be abundant in the literature were not, including employment changes for PWD (*n* = 3) (41–43), as well as instances where PWD were included in pandemic decision-making (*n* = 0).

In the included articles, it is important to address that telehealth exists as both a barrier and a facilitator to PWD during this pandemic. This technology can add both significant advantages, such as eliminating the need for travel (38, 46, 60, 64), as well as important disadvantages, such as difficulty of use for some PWD (32, 34, 54, 58, 69, 70). Researchers note that telehealth is effective for treatment and diagnosis of PD, MS and pediatric feeding disorders (79–81, 83). However, many articles cited unavoidable difficulties when utilizing telehealth for diagnosis and treatment of chronic pain, hearing impairments and individuals with symptoms of psychosis (54, 58, 69, 70). Furthermore, telehealth may be difficult to use if the patient does not have high-speed internet or a helpful family member to assist with calls. Researchers Chang and Lipner (32), as well as McKee et al. (34) note the importance of sign-language interpreters wherever needed for PWD during virtual appointments. It is imperative for health care professionals to discuss and plan with each patient that has a disability about the aforementioned potential telehealth issues, to ensure appropriate accommodations. Researchers and health care professionals should examine the telehealth application reviews by Miele et al. (80) and Moccia et al. (81) for useful telehealth strategies and tools to implement this technology with different PWD.

In the New Innovation subtheme, it was promising to see that several individuals, businesses and governments identify inclusivity issues and provide inventive solutions (32, 34, 55, 85–87). Certain innovations, such as virtual exercise programs, may be even more convenient, affordable and successful for PWD than traditional in-person methods (86). The examples presented in this subtheme may provide inspiration for others to implement the same or similar innovations in their region and potentially reach out to lesser served rural areas. Several of the included articles present clear issues that still require novel solutions (28). For example, the global use of innovative tools such as alternative text, descriptive video and proper color contrasting could increase accessibility of COVID-19-related information for individuals with visual impairments or blindness (28). This innovation would be helpful not only during a pandemic but would likely facilitate accessibility for this population for any future public health communications.

When discussing PWD, who are often marginalized, it is important to address the potential for intersectionality (93). The theory of intersectionality proposes that social inequality, discrimination and social hierarchy can be influenced by several aspects of one's identity, such as race, gender and disability, thereby creating a situation in which marginalized populations experience increased inequalities when several aspects of their identities intersect (93). For example, researchers Kolakowsky- Hayner and Goldin (48) describe how disability and gender can interact to create adverse outcomes. Specifically, the authors explain how among individuals with acquired brain injuries (ABI), women are more likely to experience disadvantageous financial situations, and increased difficulties accessing essential services and regular care (48). Here, it is clear that being a person with a disability and being a woman, both of which are aspects of identity that commonly are associated with inequality intersect to create an exponentially detrimental situation for the individual (94). Similarly, Kolakowsky-Hayner and Goldin (48) also identify an increased risk for women and members of the LGBTQ+ community to experience intimate partner violence during the pandemic. Lund (40) also details how stay-at-home orders can make it very difficult to report this violence, even when using telehealth calls, as many PWD may require a family member to assist with the appointment. During virtual appointments, health care professionals should be aware of these risks and acknowledge the possibility that the patient's family member may be their perpetrator. Wherever possible, health care professionals should ask caregivers to leave the room to provide privacy and a consequent opportunity for abuse reporting.

### Implications for Health Care Professionals and Government Officials

This review highlighted several implications for health care professionals. Firstly, accessible information is imperative for PWD to be informed regarding a crisis, and to be able to make the appropriate risk management decisions (28, 71). Governments have a responsibility to ensure accurate and accessible information is being presented at press conferences and on government websites. Researchers identify the need for organizations to portray important information in a simpler and easier-to-understand manner (26, 27). Specifically, Goggin and Ellis (26) emphasize the importance of using “easy English,” which they describe as, “using common words, simple sentence structure and using meaningful images to support information.” Guidry-Grimes et al. (27) make similar recommendations using what they term “simple English.” This method can make it easier for individuals with IDDs to understand complex health information such as COVID-19 details. Furthermore, in many public and health care settings around the world, there are little to no accommodations provided for people whose communication abilities have been hindered. This, as noted by McKee et al. (34), is in direct violation of the Americans with Disabilities Act. Clear masks, speech recognition applications and educational assistants must be provided to individuals who are DHH in order to improve communication.

### Limitations

This study was limited by the type of articles included. Many eligible articles did not contain evidence-based information but rather detailed experiences and opinions from the research experts in the form of editorials, opinion and commentary articles. While this literature did expose the authors to new and important information, evidence included from commentary and opinion articles are not as accurate and robust as scientific studies. Furthermore, our study was limited to articles written in English and many originated from Western, developed countries. The information from developing countries reveals that PWD in those locations are likely more at risk of experiencing negative impacts from the pandemic. Thus, it will be imperative for researchers to carefully examine developing countries' experience with COVID-19 and disability and how this may be different from developed countries. Finally, because the articles included were all published before September 22, 2020, the results of this study may be limited to the first wave of the pandemic and do not reflect additional barriers and facilitators that might emerge over the long-term (95).

### Implications for Future Research

The number of themes (*n* = 8) that emerged in this review highlight the importance of this topic, the barriers PWD face and some areas that may need more research. For example, telehealth, access to information and mental health were commonly mentioned in the recent literature, while topics such as domestic violence and financial impacts on PWD seemed under-researched. These neglected topics should be emphasized during future research in this area, in order to better comprehend the overall impact of the pandemic response on PWD. Additionally, as more data regarding inclusive policy successes is conducted, a review on this area could lead to a wider uptake of inclusive policies globally. As previously mentioned, there is a gap to be filled by answering our research question in other languages and in a wider range of countries to better understand the impacts of COVID-19 for PWD on a global scale. Finally, the authors originally expected to identify instances of inclusion of PWD in the pandemic response decision-making process, but unfortunately, no articles mentioned such occurrences. Upcoming research should investigate whether this occurred in any circumstances and if so, compare instances in which PWD were consulted to instances in which PWD were excluded from decision-making.

## Conclusion

This scoping review examined 74 articles and the analysis exposed several significant barriers and facilitators for the daily lives of PWD. Significant barriers in the daily lives of PWD during the pandemic included: access to information, ease of communication, financial impacts, mental health impacts, access to essential services, physical safety, educational challenges, and changes to care and rehabilitation. The noted facilitators for daily life of PWD included: changes to care and rehabilitation, new innovations, social and familial support and inclusive policy measures.

The results of this study reveal that pre-pandemic issues that were already barriers for PWD in their daily lives, such as access to inclusive care, financial barriers and communication issues, were exacerbated by the current pandemic. Furthermore, new challenges, such as access to COVID-19-related information and challenges regarding mandatory telehealth, were presented to PWD during the pandemic. Therefore, this review provides insight into the variety of inequalities still pervasive worldwide. Alternately, there were several examples of technological and policy innovation that attempted to solve inclusivity issues for PWD. These results have the potential to inspire further changes and solutions to issues that PWD are facing, in the context of COVID-19 and beyond. Furthermore, the information presented can inform policy decisions, and provide future researchers with the groundwork for more detailed investigations into the topics presented.

## Author Contributions

SC and SF database search, screening and selection of articles, analysis and interpretation, and final write-up. Both authors contributed to the article and approved the submitted version.

## Funding

The publication of this article has been supported by the University of Ottawa scholarly communication support.

## Conflict of Interest

The authors declare that the research was conducted in the absence of any commercial or financial relationships that could be construed as a potential conflict of interest.

## Publisher's Note

All claims expressed in this article are solely those of the authors and do not necessarily represent those of their affiliated organizations, or those of the publisher, the editors and the reviewers. Any product that may be evaluated in this article, or claim that may be made by its manufacturer, is not guaranteed or endorsed by the publisher.
